# Analysis of patient's willingness and concerns for discharge following shoulder arthroplasty

**DOI:** 10.1016/j.jseint.2021.12.015

**Published:** 2022-02-03

**Authors:** Kevin M. Magone, Erel Ben-Ari, Dan Gordan, Yaniv Pines, Michael A. Boin, Young W. Kwon, Joseph D. Zuckerman, Mandeep S. Virk

**Affiliations:** a Division of Shoulder and Elbow Surgery, Department of Orthopedic Surgery, NYU Grossman, School of Medicine, NYU Langone Orthopedic Hospital, NYU Langone Health, New York, NY, USA; b Division of Orthopaedic Surgery, Tel-Hashomer “Sheba” Medical Center, Sackler School of Medicine, Ramat Gan, Israel

**Keywords:** Shoulder, Shoulder arthroplasty, Patient discharge, Patient disposition, Discharge barriers, Length of stay

## Abstract

**Background:**

Patient's willingness and barriers for discharge after shoulder arthroplasty (SA) has not been studied. The aim of this study was to prospectively analyze patient's willingness for discharge and barriers to discharge beyond postoperative day #1 (POD#1) after SA.

**Methods:**

In this prospective study, patients undergoing primary or revision SA (anatomic, reverse, or hemiarthroplasty) at our institution were enrolled to determine their willingness and concerns for discharge after SA. Patient's willingness for discharge was inquired daily until discharge. Demographic information, patient's medical history, intraoperative details (duration of surgery, estimated blood loss, intraoperative complication), discharge disposition, length of stay (LOS), and reasons for extension of LOS beyond POD#1 were analyzed.

**Results:**

A total of 184 patients who underwent SA were included. Eight patients were discharged on POD#0, 114 patients on POD#1, 37 patients on POD#2, and 25 patients after POD#2. One hundred nineteen (119) patients were discharged to home, 40 were discharged to home with services, 15 were discharged to nursing facilities, and 10 were discharged to rehabilitation centers. Reasons for extension of LOS past POD#1 included patients failing to clear home safety evaluation (n = 4), inadequate pain control (n = 6), worsening of preexisting medical conditions (n = 8), delay in patient disposition (awaiting placement in a rehabilitation facility [n = 6] and awaiting culture results [n = 9]). Social reasons (n = 29) were the most common reasons for extension of LOS. These included patients requesting an extra day of stay (n = 20), patients requesting rehabilitation facility placement (n = 5), lack of a timely ride home (n = 2), and family-related reasons (death in the family [n = 1], lack of home help [n = 1]).

**Conclusions:**

This prospective study demonstrates modifiable factors associated with LOS beyond POD#1 (inadequate pain control, logistic delays in disposition, and patient-related social concerns) after SA. With increasing interest in same-day discharge and rising concerns to control cost and use bundled payment initiatives with SA, improving patient's willingness to discharge by addressing their concerns can improve early discharge after SA.

One of the goals of postoperative care after a surgical procedure includes appropriate patient discharge either to home or a health-care facility. With the introduction of newer perioperative pain control techniques, optimized rehabilitation protocols, appropriate patient selection, and perioperative care, length of stay (LOS) after major orthopedic procedures has considerably decreased in the last few decades with more to home discharges.^5^^,^^9^^,^^10^ Furthermore, the introduction of bundled payments that incentivize care providers and hospitals for reducing LOS without affecting patient satisfaction has further reduced the LOS after joint replacement.^5^ Reducing the LOS after shoulder arthroplasty (SA) also has the advantage of decreasing the overall cost of patient care and reduces patient exposure to the hospital environment.^2^^,^^5^^,^^9^

Additionally, the presence of dedicated rehabilitation protocols, streamlined perioperative care, and strict disposition guidelines has decreased discharge to extended care facilities and nursing homes, which are also an added cost to the health-care system.^9^ Currently, nonhome discharges for hemiarthroplasty (HA), anatomic and reverse total SA for elective indications, and proximal humerus fractures range from 11.5% to 21.5%.^6^^,^^8^^,^^10^ The majority of factors studied so far associated with extended LOS after SA have been identified in retrospective studies and are largely nonmodifiable, which include patients age, medical comorbidities, socioeconomic status, insurance type, and type of SA.^2^^,^^5^^,^^7^, ^8^, ^9^, ^10^ Menendez et al recently evaluated extended LOS for elective anatomic and reverse SA and reported an incidence of 24.8%.^8^ However, the study results were limited because of the retrospective study design, and the patient's true willingness and barriers for discharge were not captured because of lack of documentation in the medical record.

Reducing the LOS after SA should not compromise patient satisfaction and must address patient concerns related to discharge. With the growing trend of outpatient SA and a climbing volume of SA procedures, a thorough understanding of patient concerns and willingness for a same or next-day discharge after SA is necessary. Therefore, the aim of this study was to prospectively analyze patient's willingness for discharge and investigate barriers or reasons for extension of LOS beyond postoperative day 1 (POD#1) after SA. The hypothesis for this study is the majority of patients undergoing SA procedures will be discharged on the same or next day following the procedure.

## Materials and methods

Following institutional review board approval, a prospective cohort study was conducted to evaluate patient's willingness and concerns for discharge after SA. All patients undergoing primary or revision anatomic (aTSA), reverse (rTSA) total SA, and HA at our institution were prospectively enrolled in the study. All etiologies (arthritis, avascular necrosis, cuff arthropathy, dislocation arthropathy, irreparable rotator cuff tear, fracture, infection, aseptic loosening, mechanical failure, prosthetic instability) for primary or revision SA were included. All procedures and etiologies were included to acquire a greater understanding of patient's willingness and barriers to discharge rather than excluding higher risk procedures for extended LOS (ie, revision procedure for infection).

All patients had preoperative counseling regarding what to expect after surgery, including but not limited to the LOS, pain requirements, sling use, physical therapy, home care assistance, and follow-up. All surgeries were performed through a standard deltopectoral incision using two shoulder systems (DJO Surgical, Dallas, TX, USA; Exactech Inc., Gainesville, FL, USA). Surgeries were performed under regional anesthesia (interscalene block) and intravenous sedation unless the anesthesiologist determined that a patient required additional general anesthesia with endotracheal intubation because of medical comorbidities. All patients received an interscalene block under ultrasound guidance with a long-acting local anesthetic (bupivacaine 0.5%) as well as standardized institutional postoperative multimodal pain regimen for SA; all patients received orders of Tylenol Johnson & Johnson Inc., New Brunswick, NJ; (1000 mg Q 8 hours) and nonsteroidal anti-inflammatory drugs (ibuprofen 600 mg Qhr) for mild pain. Patients with moderate pain (Visual Analog for Pain score 6-8) were given tramadol (25 mg)/oxycodone (5 mg). Patients with severe pain were given tramadol (100 mg)/oxycodone (10 mg). If patients had allergy to any of the aforementioned medications, appropriate substitutions were made. Pain management specialists were involved when there was need for lidoderm patches, for opioid-dependent patients, patients on suboxone therapy, or other special scenarios. Neuropathic medications like gabapentin or Lyrica (Pfizer Inc., New York, NY) were only started at the discretion of pain management specialists.^3^

Postoperatively, the patient's arm was placed in a sling, and occupational and physical therapies were started on the day of surgery. All surgeries in this study were performed in a hospital setting.

The decision for discharge was made after discussion with the surgical attending, rehabilitation team, and medical hospitalist/subspecialist. Patient's willingness for discharge and concerns related to discharge were inquired by the shoulder and elbow fellow (twice daily; AM and PM) until discharge. The fellow asked the same standard question to all patients, “Do you feel comfortable going home?” The patient's response was documented in the medical chart, along with patient's concerns. Subsequent evaluations were performed by the fellow to determine the resolution of previous concerns and inquire about any new concerns related to discharge and documented in patient's chart. In cases where the physician (surgeon or medical attending) or rehabilitation team recommended extension of the inpatient stay, these cases were documented as well in the medical record. These reasons fell into the following categories: treatment of preexisting medical condition(s) or medical complication, patient determined to be unsafe for home discharge by the therapist, and administrative or logistic delays in discharge (rehabilitation or facility placement, peripherally inserted central catheter placement delay, awaiting final culture results, delay in home services).

Demographic information, patient's medical history, Charlson Comorbidity Index (CCI) score, intraoperative details (duration of surgery, estimated blood loss, intraoperative complication), discharge disposition, LOS, reasons for extension of LOS beyond POD#1, and readmissions within 90 days were collected. Standard descriptive summaries (means, standard deviations, percentages) were utilized to analyze the data.

### Statistical analysis

Subgroup analysis was performed between the patients in the early discharge (POD#1 or less) and extended LOS (POD#2 or more) groups to evaluate the risk factors and 90-day readmission rate. A multivariate regression analysis was conducted to identify risk factors for extended LOS. The Pearson Chi-Square (χ^2^) test was used for categorical (ambulatory status, living alone, revision arthroplasty, CCI, and insurance type) variables, and the t-test for equality of means was used for continuous (age) variables. A *P* value < .05 was considered statistically significant. All statistical analyses were done using Graph Pad Prism 5 (Graph Pad, LaJolla, CA, USA) and stored using the Excel software (Microsoft Corporation, Richmond, WA, USA).

## Results

### Demographics

From December 2019 to October 2020, 184 patients undergoing SA procedures were prospectively enrolled for this study. Of the 184 procedures, 162 (88%) were primary shoulder arthroplasties, and 22 (12%) were revision shoulder arthroplasties (Table I). Of the primary procedures, 36 (22%) were aTSA, 125 (77%) were rTSA, and 1 (1%) was a HA. Of the revision procedures, 22 (100%) were rTSA. The average age of the patient cohort was 69 years (range, 39 – 92 years), and 93 (51%) were female (Table I).

**Table I:** Patient demographics.

Average age (range; yr)	69 (39 – 92 yr)
Gender (number of patients [%])	Female (93 [51%]) Male (91 [49%])

Arthroplasty type	Number of patients (%)
Primary arthroplasty	162 (88)
Reverse	125/162 (77)
Anatomic	36/162 (22)
Hemiarthroplasty	1/162 (1)
Revision arthroplasty	22 (12)
Reverse	22/22 (100)
Total arthroplasty cases	184 (100)

### Discharge disposition

One hundred and nineteen (119/184, 65%) patients were discharged home, 40 (22%) patients were discharged home with services, 15 (8%) patients were discharged nursing facilities, while 10 (5%) were discharged rehabilitation centers (Table II). The majority (92/122, 75%) of patients did not live alone and consequently had home support (spouse, other family member, care giver). Only 6 of the 122 (5%) patients in the early discharge group and 6 of the 62 (10%) patients in the extended LOS group had difficulties ambulating and used a walker, rollator, or a wheelchair. Patients in the extended LOS group were older, and a higher percentage of these patients were in the severe CCI category than early discharge group.

**Table II:** Length of stay (LOS) and discharge destination after shoulder athroplasty.

Discharge after surgery (postoperative day [POD])	Number of patients (%)
POD#0	8 (4)
POD#1	114 (62)
POD#2	37 (20)
POD# > 2	25 (14)
Total	184 (100)

Discharge destination	Number of patients (%)
Home without services	119 (65)
Home with services (nursing, therapist, home aide)	40 (22)
Nursing facility	15 (8)
Rehabilitation center	10 (5)
Total	184 (100)

### Patient willingness/concerns for discharge and LOS

One hundred twenty-two of 184 (66%) patients expressed willingness to be discharged on POD#0 (8) or POD#1 (114). Three of the patients who were discharged on POD#0 requested same-day discharge as a precaution to prevent contracting the coronavirus infection (COVID-19). There were 62 (34%) patients who were discharged on POD#2 (37) or after POD#2 (25), which was defined as an extended LOS for this study (Table II). The most common patient concern against discharge on POD#1 was “not feeling good or feeling weak” and therefore requested an additional day stay even though they were determined to be medically cleared for discharge. Five patients (n = 5) requested rehabilitation or nursing facility placement due to lack of home assistance. Two (n = 2) patients did not have a ride home on the day of discharge, one patient did not have home health aide available on the day of discharge, and in one case, the family requested an additional day of stay for the patient due to death in the family (Table III).

**Table III:** Reasons associated with increased length of stay (LOS) beyond first postoperative day (>POD#1) after shoulder replacement.

Causes for LOS > POD#1 day	Number of patients	Percentage
Patient failed home safety evaluation	4	6
Insufficient pain control	6	10
Worsening medical conditions	8	13
Acute blood loss anemia (requiring 1U packed red blood cells)	1	
Hypotension	1	
Hyperglycemia	1	
Phrenic nerve palsy with persistent dyspnea	1	
Persistent nausea	1	
Shortness of breath	1	
Asthma exacerbation	1	
Small bowel obstruction (no surgical intervention needed)	1	
Logistic delays in disposition	15	24
Waiting for extended care facility placement (despite medical and surgical clearances)	6	
Waiting for finalization of culture results (revision shoulder arthroplasty) and coordination of home services	9	
Social reasons	29	47
Patient requesting an extra night stay	20	
Patient requesting placement in a rehabilitation facility	5	
Family members not available for pick up (transportation to home)	2	
Death in family	1	
Health aide unavailable on the day of discharge	1	
Total	62	100

*POD*, postoperative day.

Apart from the aforementioned social reasons (n = 29, 47%), other reasons for extension of LOS > POD#1 included administrative or logistic delays in patient disposition (n = 15, 24%), treatment of a medical condition or complication (n = 8, 13%), inadequate control of shoulder pain (n = 6, 10%), and patients not cleared by rehabilitation team due to increased risk of fall at home secondary to lower extremity ambulation issues (lack of progression with therapy; n = 4, 6%). Delay in patient disposition (n = 15, 24%) included waiting for rehabilitation facility approval (n = 6) and waiting for intraoperative cultures to finalize for home antibiotic plan and coordination of home services (n = 9).

Eight (n = 8, 13%) patients had inpatient stay beyond POD#1 because they required treatment of their medical condition or a complication, which included acute blood loss anemia requiring a blood transfusion (n = 1), persistent hypotension (n = 1), asthma exacerbation (n = 1), hyperglycemia (n = 1), phrenic nerve palsy and dyspnea (n = 1), persistent nausea (n = 1), shortness of breath (n = 1), and small bowel obstruction (n = 1). The patient with a small bowel obstruction did not require any surgical intervention (Table III).

### Comparison of early LOS and extended LOS groups

Subgroup analysis was performed between the patients discharged before POD#1 (shorter LOS) and patients discharged after POD#1 (extended LOS) with respect to patient's ambulatory status, living status at home (alone vs. with support), patients' age, CCI score, type of insurance (medicare, medicaid, private), and revision arthroplasty (vs. primary arthroplasty) (Table IV). None of the aforementioned risk factors including revision SA (vs. primary arthroplasty, *P* = .22), age at time of surgery (mean age, 72 vs. 68 years, *P* = .94), and CCI (severe CCI category 31/62 [50%] vs. 29/122 [24%], *P* = .15) were found to be statistically significant for extension of LOS > POD#1 on a multivariate analysis (Table IV).

**Table IV:** Multivariate analysis of risk factors for a short LOS (POD#<1) compared with extended LOS (POD#≥2) following shoulder arthroplasty.

Risk factor	N	Short LOS (POD#<1) n/122 (%)	Extended LOS (POD#≥2) n/62 (%)	*P* value
Age (yr)	184	68 (range, 39-86)	72 (range, 45-92)	.94
Living alone	54	31/122 (25%)	23/62 (37%)	.36
Ambulation difficulties (walker/rollator/wheelchair)	12	6/122 (5%)	6/62 (10%)	.21
Revision arthroplasty	22	12/122 (10%)	10/62 (16%)	.22
Insurance type				
Medicare	116	72/122 (59%)	44/62 (71%)	
Medicaid	8	3/122 (2%)	5/62 (8%)	.09
Private	60	47/122 (39%)	13/62 (21%)	
Total	184	122/122 (100%)	62/62 (100%)	
CCI category				
Mild (CCI = 1-2)	56	44/122 (36%)	12/62 (19%)	
Moderate (CCI = 3-4)	68	49/122 (40%)	19/62 (31%)	.15
Severe (CCI ≥ 5)	60	29/122 (24%)	31/62 (50%)	
Total	184	122/122 (100%)	62/62 (100%)	

*POD*, postoperative day; *LOS*, length of stay; *CCI*, Charlson Comorbidity Index; *N*, total number of patients in the risk factor category; *n*, number of patients.

Overall, there were 9 readmissions within 90 days in both groups of patients. Readmissions were almost equally split between the two groups, as there were 5 readmissions in the early discharge group (subscapularis tear [1], cellulitis [1], shoulder dislocation [2], surgical wound dehiscence [1]) and 4 readmissions in the delayed LOS group (low hemoglobin level [1], glenoid loosening [1], deep wound infection [1], lack of home support [1]).

## Discussion

With a large, expected need for SA in the near future and rising popularity of outpatient shoulder arthroplasties, a thorough assessment of patient willingness and concerns for discharge is essential to improve patient satisfaction and safety with expedited discharges. This prospective study reports the patient's willingness and concerns for discharge and reasons for extended inpatient stay beyond the first POD after SA. We found that majority of patients are discharged on POD#1 after SA, and majority of the patient-related concerns/factors for extended inpatient stay after POD#1 are modifiable and can be improved to reduce the overall LOS.

At our institution, the vast majority of the patients (122/184, 67%) who underwent a SA procedure (primary or revision; aTSA, rTSA, or HA) were discharged home with or without home services on POD#0 or POD#1. However, one-third (62/184, 33%) of the patients were discharged on or after POD#2. A large percentage of the patient concerns or reasons for discharge beyond POD#1 in our study were social in nature (47%). This is in contrast to findings of previous studies in which postoperative pain was reported to be the primary factor associated with extended LOS.^1^^,^^4^^,^^7^ In our series, inadequate pain control as a reason for stay beyond POD#1 was seen in a very small percentage of the cohort and is attributed to superior perioperative pain control with interscalene block (longer acting agent bupivacaine) and postoperative multimodal analgesia. Additionally, prior retrospective studies identified largely nonmodifiable risk factors for extended LOS such as patient's gender, age, race, insurance type, medical comorbidities, diagnosis, operative time, and a provider's case volume.^1^^,^^4^^,^^7^ In this prospective study, we conducted a multivariate analysis of previously determined major risk factors including patients' age, ambulatory difficulties, lack of home support (living alone), revision arthroplasty, medical comorbidities (CCI), and insurance type for an extended LOS but did not find any statistical significance for these risk factors to be associated with LOS beyond POD#1. The prospective nature of our study allowed us to look at these potential risk factors closely and also determine patient's concern and willingness, which has not been reported previously.

This study is important because it unveils modifiable patient-related factors and administrative/logistic delays that can be effectively corrected to decrease the LOS after inpatient SA. Modifiable reasons for an extended LOS in this study accounted for 77% of the reasons for extension of LOS beyond POD#1 and included social reasons (requests for an extra night and rehabilitation facility placement, and lack of a timely ride home), logistic delays in disposition, and inadequate pain control. These factors can be mitigated by preoperative patient counseling and addressing patients' concerns by providing resources (medical cab, emergency home services, perioperative pain control) and therefore expedite discharge from hospital after SA. Other less modifiable reasons for an extended LOS included treatment of worsening medical conditions, lack of progression with therapy, and other social reasons (family death and living alone), which can be addressed with preoperative optimization of medical conditions, “prehabing” with home exercise program, and preoperative counseling regarding the LOS with the patient.

In a retrospective review of 415 elective TSAs, Menendez et al reported the most common reason for an extended LOS to be pain control (41%) followed by worsening medical conditions (39%), limited social support (18%), and blood transfusions (2%).^8^ In contrast to the study by Menendez et al, our study had a small percentage of patients with inadequately controlled postoperative pain that required extended hospital stay, and we believe that regional anesthesia with a long-acting interscalene block and multimodal postoperative analgesia were the reason for this finding. Furthermore, patients with extended stay due to worsening of medical condition were considerably less in our study (13%) than those in the study by Menendez et al (39%).^8^ However, our study had higher proportion of patients staying beyond POD#1 due to social reasons (47% vs. 18%).^8^ This is an important finding as we were able to investigate these reasons prospectively without any recall bias as was present in prior retrospective studies.^2^, ^3^, ^4^, ^5^^,^^7^, ^8^, ^9^, ^10^ At our institution, following the results of this study, all patients undergoing SA are offered a social work consult preoperatively, and social barriers are identified in an effort to minimize its impact on LOS.

This study has limitations that should be considered when interpreting the results. First, the number of patients in this study is less than that in previously reported retrospective studies.^8^ The strength, however, of our study is that it prospectively evaluated patients' willingness and concerns for discharge as we were able to elicit specific reasons (provider and patient-related) for extended LOS as opposed to a retrospective chart review. Second, we did not stratify patients according to their discharge preference/capabilities preoperatively. Preoperative prediction of patients' discharge disposition can effectively influence patient's LOS following SA. Therefore, following the results of our study, all patients undergoing SA in our institute are offered a social work consult preoperatively, and social barriers are identified to minimize its impact on LOS. Third, the external validity of the study results may be affected by practice setting as this study took place in a large, urban cosmopolitan setting, and specific social reasons documented may not be relatable or applied to all practice settings in different regions of the country (as with a request for another night stay to avoid high traffic volumes when traveling home). Furthermore, near-exclusive use of regional anesthesia with nerve blocks and multimodal pain regimen allowed superior pain control compared with what was reported in previous studies. Fourth, for the purpose of this study, the term extended LOS was used for patients discharged after POD#1, which is different compared with previously reported studies and what is approved by the Center for Medicare Services.^4^^,^^7^^,^^8^ POD#1 discharges after joint replacements are considered expedited discharges, and we were interested in knowing the risk factors associated with inpatient stay after POD#1. Finally, a portion of this study was conducted during the COVID-19 pandemic. During this time, the patient may have elected to have shorter LOS including same-day discharge due to fear of contracting the virus.

## Conclusion

This prospective study identifies modifiable factors associated with LOS beyond POD#1 (inadequate pain control, logistic delays in disposition, and patient-related social concerns) after SA. With increasing interest in same-day discharge and rising concerns to control cost and utilize bundled payment initiatives with SA, improving patient's willingness to discharge by addressing their concerns can improve early discharge after total SA. Additionally, with improved perioperative pain control, postoperative pain following SA is a less common reason for extended LOS after SA.

## Disclaimers

Funding: No funding was disclosed by the authors.

Conflicts of interest: Young W. Kwon Has consulting relationship with DJO Surgical. Mandeep S. Virk Has a consulting relationship with Exactech Inc. Joseph D. Zuckerman has stock or stock options in Apos Therapy, Inc. and Hip Innovation Technology; is a Musculoskeletal Transplant Foundation Board Member; receives publishing royalties from SLACK Inc., Thieme Inc., and Wolters Kluwer Health, is a design surgeon in Exactech, and receives royalties from Exactech. The other authors, their immediate families, and any research foundation with which they are affiliated have not received any financial payments or other benefits from any commercial entity related to the subject of this article.
